# Selection of Reference Genes for qRT-PCR Analysis of Gene Expression in *Stipa grandis* during Environmental Stresses

**DOI:** 10.1371/journal.pone.0169465

**Published:** 2017-01-05

**Authors:** Dongli Wan, Yongqing Wan, Qi Yang, Bo Zou, Weibo Ren, Yong Ding, Zhen Wang, Ruigang Wang, Kai Wang, Xiangyang Hou

**Affiliations:** 1 Key Laboratory of Grassland Resources and Utilization of Ministry of Agriculture, Institute of Grassland Research, Chinese Academy of Agricultural Sciences, Hohhot, China; 2 College of Life Sciences, Inner Mongolia Agricultural University, Hohhot, China; Texas Technical University Health Sciences Center, UNITED STATES

## Abstract

*Stipa grandis* P. Smirn. is a dominant plant species in the typical steppe of the Xilingole Plateau of Inner Mongolia. Selection of suitable reference genes for the quantitative real-time reverse transcription polymerase chain reaction (qRT-PCR) is important for gene expression analysis and research into the molecular mechanisms underlying the stress responses of *S*. *grandis*. In the present study, 15 candidate reference genes (*EF1 beta*, *ACT*, *GAPDH*, *SamDC*, *CUL4*, *CAP*, *SNF2*, *SKIP1*, *SKIP5*, *SKIP11*, *UBC2*, *UBC15*, *UBC17*, *UCH*, and *HERC2*) were evaluated for their stability as potential reference genes for qRT-PCR under different stresses. Four algorithms were used: GeNorm, NormFinder, BestKeeper, and RefFinder. The results showed that the most stable reference genes were different under different stress conditions: *EF1beta* and *UBC15* during drought and salt stresses; *ACT* and *GAPDH* under heat stress; *SKIP5* and *UBC17* under cold stress; *UBC15* and *HERC2* under high pH stress; *UBC2* and *UBC15* under wounding stress; *EF1beta* and *UBC17* under jasmonic acid treatment; *UBC15* and *CUL4* under abscisic acid treatment; and *HERC2* and *UBC17* under salicylic acid treatment. *EF1beta* and *HERC2* were the most suitable genes for the global analysis of all samples. Furthermore, six target genes, *SgPOD*, *SgPAL*, *SgLEA*, *SgLOX*, *SgHSP90* and *SgPR1*, were selected to validate the most and least stable reference genes under different treatments. Our results provide guidelines for reference gene selection for more accurate qRT-PCR quantification and will promote studies of gene expression in *S*. *grandis* subjected to environmental stress.

## Introduction

*Stipa grandis* P. Smirn. is a C3 perennial bunchgrass [1] that is distributed widely in eastern Eurasian steppes and the middle Eurasian steppe zone [2]. This species is dominant in the typical steppe of the Xilingole Plateau of Inner Mongolia [1]. The *S*. *grandis* steppe acts as a natural green barrier and plays an important role in sandstorm prevention [3]. To date, studies of *S*. *grandis* have mostly focused on ecological aspects, such as the relationship between the photosynthetic capacity of *S*. *grandis* and the soil moisture gradient [4], the effects of grazing intensity and duration on the biomass of *S*. *grandis* [5], and the effects of global warming and elevated CO_2_ on plant growth [6]. The physiological responses of *S*. *grandis* to high temperature and drought have also been reported [2]. Several genetic diversity studies have been carried out on *S*. *grandis* related to grazing and location [1, 3, 7]. Transcriptome analysis was carried out to reveal the gene expression profiles of overgrazed and non-grazed *S*. *grandis* in a typical grassland; the results showed that many stress-related genes had differential expression under long-term grazing conditions [8]. However, the mechanism underlying *S*. *grandis*’s responses to grazing and related stresses remain unclear. To further understand the stress response mechanism of *S*. *grandis*, details of the expression patterns of more stress-related genes are necessary.

Quantitative real-time reverse transcription PCR (qRT-PCR) is an efficient tool for expression analysis because of its high-throughput, accuracy, sensitivity, and ease of use compared with traditional methods, such as northern blotting and semi-quantitative reverse transcription-PCR [9–11]. The inevitable errors or experimental deviations caused by sample preparation and data analysis can affect the reliability of the results; therefore, it is essential to normalize the data using appropriate internal reference genes [12]. The stability of the reference gene’s expression is crucial for the accurate normalization of target gene expression following qRT-PCR quantification [13]. Extensive studies on reference gene stability and selection have been reported in various plants for different tissues and development stages, and under diverse stresses [14–22]. Traditionally, reference genes such as 18S *rRNA*, *GAPDH* (glyceraldehyde-3-phosphate dehydrogenase), *ACT* (β or γ actin), *TUB* (α or β tubulin), *EF1α* (elongation factor 1α), and *UBQ* (poly-ubiquitin) have been used most frequently as internal control genes [13, 17]. Recently, taking advantage of a variety of databases comprising data from microarrays, expressed sequence tags, and transcriptomes, other stably expressed genes have been used as novel reference genes for qRT-PCR, such as *CUL* (Cullin), *UBCP* (ubiquitin carrier protein) [17], *CAP* (adenylyl cyclase-associated protein), *SKIP1* (ASK-interacting protein 1) [22], *F-BOX* (F-box/kelch-repeat protein) [23], *PP2A* (protein phosphatase 2A) [24], *SAMDC* (s-adenosyl methionine decarboxylase) [12, 25], and *SNF* (sucrose non fermenting-1 protein kinase) [26]. However, the transcript levels of these reference genes vary among different conditions to some extent [20, 27]. Thus, the selection of ideal reference genes to normalize target gene expression is important to improve the reliability of qRT-PCR results.

In this study, 15 candidate reference genes that could be used potentially as internal control genes to normalize gene expression were selected. The expression stabilities of these reference genes under various abiotic stresses and three hormone treatments were assessed using four algorithms (geNorm, NormFinder, Bestkeeper, and RefFinder). According to our analysis, we were able to recommend the most suitable reference genes to quantify gene expression in *S*. *grandis* under various experimental conditions.

## Materials and Methods

### Plant materials and growth conditions

*S*. *grandis* seeds were collected from the Baiyinxile Livestock Farm of Xilinhot, Inner Mongolia (116°40ʹE, 43°33ʹN). The seeds were sown in pots filled with a soil mixture (rich soil: vermiculite, 1:1, v/v), and incubated in a growth chamber at 23°C with a 16-h light/8-h dark cycle.

No specific permits were required for the described field studies.

### Hormone and abiotic stress treatments

Four-week-old plants were used in the following experiments, and the different treatment conditions were mainly achieved according to methods described previously [12, 22, 28, 29]. Before treatment with hormones, salinity, high pH, and drought, *S*. *grandis* plants were removed from the soil gently, washed clean with tap water, and then immediately subjected to the different treatments. For plant hormone, high pH and salinity treatments, the plants were dipped separately in solutions of 100 μM salicylic acid (SA), 100 μM jasmonic acid (JA), 100 μM abscisic acid (ABA), high pH (pH 10), and 300 mM NaCl. For the drought treatment, the plants were placed on filter paper and incubated in a growth chamber. For the wounding treatment, plants growing in pots were wounded using tweezers and placed in a growth chamber. For cold and heat treatments, plants growing in pots were maintained at 4°C or 42°C, respectively. Untreated plants were used as the control. Shoot samples (from four to five plants) were taken at 1, 3, 6, 12, 24, and 48 h time points for all treatments, and an additional 0.5-h time point for drought and wounding treatments, and were frozen immediately in liquid nitrogen and stored at −80°C until RNA extraction. Three independent biological experiments were performed.

### RNA isolation and cDNA synthesis

Total RNA was extracted from the samples using the Trizol regent. The purity and integrity of the RNA samples were estimated by calculating their A260/280 absorbance ratios and by agarose gel electrophoresis analysis. Total RNA (0.5 μg), pretreated with RNase-free DNase I (Takara, Dalian, China), was reverse transcribed into cDNA using an M-MLV reverse transcriptase kit (Takara) and oligo (dT) 18 primers (Takara) following the manufacturer’s instructions. The generated cDNAs were diluted 20-fold for qRT-PCR analysis.

### Reference gene selection and primer design

The sequences of the candidate reference genes used in this study were selected based on our previous transcriptome analysis [8], and included *EF1 beta*, *ACT*, *GAPDH*, *SamDC*, *CUL4*, *CAP*, *SNF2*, *SKIP1*, *SKIP5*, *SKIP11*, *UBC2*, *UBC15*, *UBC17*, *UCH*, and *HERC2*. According to these sequences (S1 Text), specific primers for qRT-PCR were designed using the Premier5 software, with a melting temperature (T) of 59–61°C, a length of 18–25 bp, an amplicon product size of 75–160 bp, and a GC content of 40–60%; the primers are listed in Table 1.

**Table 1:** Candidate reference genes and characteristics of qRT-PCR primers in *S*. *grandis*.

Gene symbol	Gene name	Primer sequences (5′-3′)	Amplicon length (bp)	Product melting temperature (°C)	Amplification efficiency (%)
***EF1beta***	*Elongation factor 1-beta*	CCATCAACGAATACGTCCAGAG CCCCAGTTCCAAGAATCCACTA	97	83.6	104.1
***CUL4***	*Cullin-4*	GTGGAAGGCGTCTGATGTGG GCTTAGCTTTTGTGCGTCGTT	140	82.1	107.3
***UCH***	*Ubiquitin carboxyl-terminal hydrolase 6*	GAAACTATCTGGTGGAGGCGACT GGTATGCTTGACCCTGATAAATGTAG	129	82.6	106
***UBC2***	*Ubiquitin-conjugating enzyme E2 2*	AACATCACGGTCTGGAACGC GTTGGTGGCTTGTTGGGATAA	113	86.4	104.2
***UBC17***	*Ubiquitin-conjugating enzyme E2-17*	TATCCGTTCAAGCCTCCAAAG GGTCGGTAAGTAGTGAGCAGATT	160	82.2	101.9
***HERC2***	*HECT domain E3 ubiquitin ligases*	ATGGGCTTCGGGAGTTTAGG GCTTATTTGGGAGACATAGTGACC	105	85.2	107.3
***SNF2***	*Sucrose non fermenting-1 protein kinase*	GCGAGCGTTGCTACAGGAGA CCTCAGTTCGAGCAGCCAAA	101	84.3	105.7
***CAP***	*Cyclase-associated protein*	AGCTCCAGAAACCTGCATCAA GGCCTCCTTCCTTCAGTCAAA	100	81.9	106.2
***SKIP1***	*ASK-interacting protein*	GGTTCTGGACCTGTTTGGCT TCTCCAGGTTCTTCAGATTAGCA	80	81.1	99.3
***SKIP5***	*ASK-interacting protein*	TGTGAGCAAAACCCTCTGGAT TTGAGCGATGAGAATGAGTCATACT	80	80.3	105.2
***SKIP11***	*ASK-interacting protein*	TTGGGGTATTGCATTCCGAG CCAGAAGGTTTGCTTCCGAT	147	86.6	102.7
***SamDC***	*S-adenosylmethionine decarboxylase proenzyme*	TGTCGTGGTTGTTGTTTTGGTT TGCTCACTGTGGTAGACGCAG	108	82.3	103.6
***UBC15***	*Ubiquitin-conjugating enzyme 15*	GACCGCTATGTTAGGAACTGCC AGAAGAGCCAAACCAATGCC	100	83.5	104.9
***GAPDH***	*Glyceraldehyde-3-phosphate dehydrogenase*	GGTATGTCCTTCCGTGTTCC CGCCTTGATAGCCTTCTTGA	105	81.5	101.4
***ACT***	*Actin-7*	GCATGAAGGTGAAGGTGGTT TCATCGTACTCTGCCTTGGA	121	84.2	103.4

### qRT-PCR analysis

The qRT-PCR was performed on a LightCycler 480 Real Time PCR system (Roche, Basel, Switzerland) with a final volume of 20 μL per reaction, as previously described [30]. Each 20-μL reaction mixture contained 5 μL of diluted cDNA, 10 μL of SYBR Premix Ex Taq II (Takara), 0.8 μL of each primer, and 3.4 μL of sterile distilled water. The thermal cycling program was 95°C for 30 s, followed by 40 cycles of 95°C for 5 s, 60°C for 30 s, and 72°C for 15 s. Melt-curve analyses were performed using a program with constant heating from 70°C to 95°C, followed by 95°C for 15 s. Each reaction was carried out in three technical replicates.

### Stability analysis of reference genes

First, three analytical tools, geNorm (version 3.5) [11], NormFinder (version 0.953) [31], and BestKeeper (version 1) [32], were used to calculate the expression stabilities of the 15 reference genes in all samples from the different treatments.

geNorm determines the most stable reference genes from a set of tested genes in a given sample set under a certain experimental condition and calculates the gene expression normalization factor via geometric averaging of all the reference genes [11]. geNorm evaluates gene expression stability by the M value, and ranks the tested genes according to their expression stability by stepwise exclusion of the gene with the highest M value. The lower the M value, the more stable the gene’s expression. The optimal number of reference genes was determined by pairwise variation (V) with a threshold of 0.15 [11]. If the value of Vn/n+1 was below 0.15, additional genes were not required for normalization. NormFinder is an algorithm based on a mathematical model of gene expression and uses a solid statistical framework to estimate the expression variation of candidate normalization genes, as well as the variation in sample subgroups, to identify the optimal normalization gene among a set of candidates [31]. A stability value, which is a direct measure of the estimated expression variation for each gene, is provided by NormFinder; thus, the systematic errors introduced during the process of normalization can be evaluated. In geNorm and NormFinder, the quantities obtained from Ct (cycle threshold) values via the delta-Ct method were used for data input, whereas Bestkeeper used raw Ct values. BestKeeper uses the standard deviation (SD) and coefficient of variance (CV) of the Ct values to calculate the stability of a candidate reference gene; the highest SD and CV values equate to the least stable reference genes [32].

Finally, RefFinder (http://fulxie.0fees.us/?type=reference), which is a comprehensive web-based tool that integrates geNorm, NormFinder, BestKeeper, and the comparative delta Ct method, was applied to determine the most suitable reference genes for the overall final ranking [33].

### Validation of reference gene expression

Peroxidase (POD/POX, EC:1.11.1.7), phenylalanine ammonia-lyase (PAL), late embryogenesis abundant protein (LEA), lipoxygenase (LOX), heat shock protein 90 (HSP90), and pathogenesis-related protein 1 (PR1) encoding genes were obtained from the *S*. *grandis* transcriptome library [8] and used to confirm the utility of the validated candidate reference genes for qRT-PCR.

The transcript levels of *SgPOD* and *SgPAL* under all treatments, *SgLEA* under drought, cold, salt, high pH and ABA treatments, *SgLOX* under wounding and JA treatments, *SgHSP90* under heat treatment, and *SgPR1* under SA treatment were quantified using the most and least stable reference genes, respectively. The primers were as follows:

*SgPOD*: TCGACTTCGCTGTTCCCACT/CCTTTTGCGGTGAAGTTTCC

*SgPR1*: GTCGTCTGCAACAACAACGG/GGGACTACTGCGAAGCAATCA

*SgPAL*: AGCAGCACAACCAGGACGTA/GTCACGCAGTTCTTGACGGA

*SgLEA*: CTTCCACTTCCATGATTTACGC/CATCCATACAGACCCCAGACAC

*SgLOX*: CTGGAGGGCATCGAGCAC/GGGTAGAGCAGCATGTAGGG

*SgHSP90*: CGGATCTTGTGAACAACCTCG/GTAGAAACCAACACCAAACTGACC

The 2^−ΔΔCT^ method [34] was used to calculate the relative expression levels. Each biological sample was analyzed using three technical replicates.

## Results

### Performance of primers and expression profiles of candidate reference genes

Fifteen fragments of candidate reference genes (Table 1), obtained from previous *S*. *grandis* transcriptome data [8], that exhibited stable expression after grazing (within a 2-fold change in expression level) were selected. Agarose gel electrophoresis and melting curve analysis showed that a specific fragment of the expected size and a single peak were observed (S1 Fig), respectively, after reverse transcriptase-PCR amplification using cDNA templates with each primer pair. The PCR amplification efficiencies of each primer pair across all samples ranged from 99.3% to 107.3% (Table 1).

**Fig 1:**
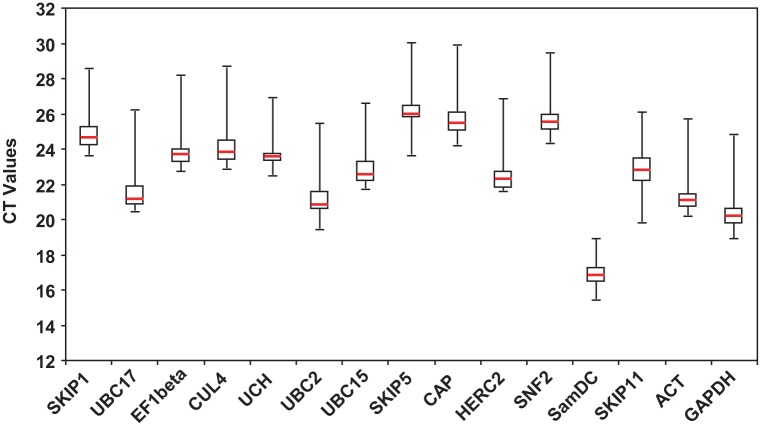
Distribution of Ct values of candidate reference genes in *S*. *grandis* plants obtained by qRT-PCR. Ct values for each reference gene are tested in all samples. Boxes represent the first and third quartiles of the data. Red lines across the boxes indicate the median Ct values. Whiskers show the maximum and minimum values.

The expression levels of the candidate reference genes were determined by qRT-PCR across all samples (including normal and different stress conditions) (Fig 1), and a wide range of Ct values, from 15.25 to 30.03, were found, with the values for most of the genes lying between 20 and 26. *SamDC* exhibited the highest expression abundance, with a mean Ct of 16.91, while *SKIP5* had the lowest expression level, with a mean Ct of 26.08 across all samples.

*SamDC* (3.40 cycles) and *UCH* (4.33 cycles) showed the smallest expression variations across all the samples. In contrast, *SKIP5* (6.34 cycles) and *SKIP11* (6.21 cycles) exhibited the largest relative expression variations among all the samples.

### geNorm analysis of reference genes

**Fig 2:**
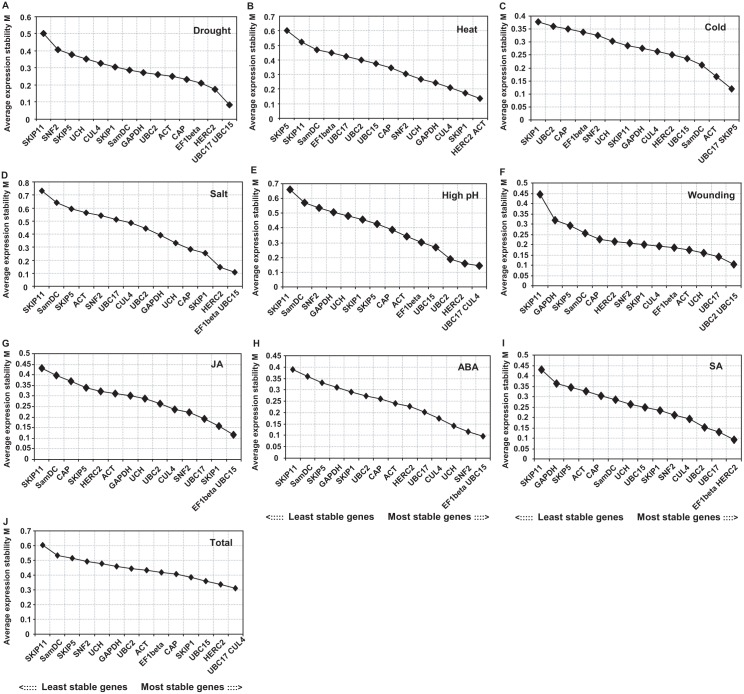
Average expression stability (M) evaluated using geNorm. M values and rankings are indicated for *S*. *grandis* under different stresses. (A) Drought treatment. (B) Heat treatment. (C) Cold treatment. (D) Salt treatment. (E) High pH treatment. (F) Wounding treatment. (G) JA treatment. (H) ABA treatment. (I) SA treatment. (J) Total.

The geNorm results are shown in Fig 2. For the drought treatment, *UBC15* and *UBC17* were the most stably expressed genes with the lowest M value of 0.0844. For ABA, salt, and JA treatments, the most stably expressed genes were *EF1beta* and *UBC15*, with M values of 0.096, 0.106, and 0.117, respectively. For heat treatment, *HERC2* and *ACT* had the lowest M value of 0.137. Under cold treatment, *UBC17* and *SKIP5* had the lowest M value, and for high pH treatment, *UBC17* and *CUL4* were the most stable genes. After SA and wounding treatments, the M values of the combinations *EF1beta* and *HERC2*, and *UBC2* and *UBC15* were the lowest, respectively. Under heat and cold treatments, the least stable genes were *SKIP5* and *SKIP1*, respectively. In the other seven treatments, *SKIP11* was the most unstable gene. Across all samples, *UBC17* and *CUL4* ranked as the most stable genes, while *SKIP11* was the least stable gene.

**Fig 3:**
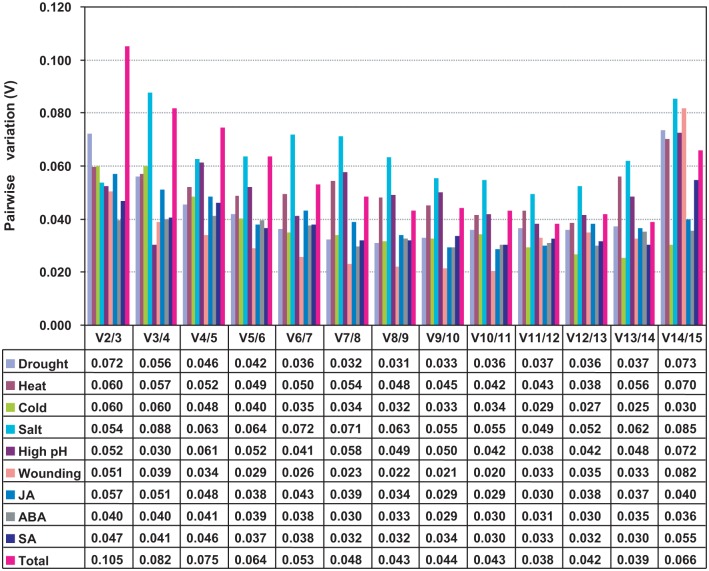
Pairwise variation (V) analysis of the reference genes in *S*. *grandis* using geNorm. The pairwise variation (Vn/n+1) between the normalization factors NFn and NFn+1 was applied to determine the optimal number of reference genes recommended to normalize qRT-PCR data in *S*. *grandis* under various stresses. If the Vn/n+1 values were below 0.15, an additional (n + 1) reference was not required.

The optimal number of reference genes for normalization was calculated by pairwise variation (V). Under various conditions, the Vn/n+1 values were lower than 0.15 (Fig 3), which suggested that adding a further reference gene would not improve the normalization quality and a combination of the two most stable reference genes was sufficient for normalization.

### NormFinder analysis of reference gene expression stability

Based on the results calculated by NormFinder (Table 2), the three highest ranked genes under ABA treatment were *CUL4*, *UBC15*, and *UBC17*; under cold treatment were *SKIP5*, *HERC2*, and *UBC17*; under heat treatment were *ACT*, *SKIP1*, and *GAPDH*; under JA treatment were *UBC17*, *EF1beta*, and *UBC15*; under salt treatment were *UBC15*, *EF1beta*, and *HERC2*; under high pH treatment were *UBC15*, *EF1beta*, and *SKIP5*; under SA treatment were *HERC2*, *UBC17*, and *EF1beta*; under wounding treatment were *UBC2*, *UBC15*, and *UBC17*; and under drought treatment were *EF1beta*, *UBC15*, and *GAPDH*. According to all the samples in the study, *UBC15*, *HERC2*, and *EF1beta* were the most stable genes. In addition, the least stable genes ranked by NormFinder and geNorm were consistent: for cold and heat stresses they were *SKIP1* and *SKIP5*, and for the other treatments it was *SKIP11*.

**Table 2:** Stabilities of candidate reference genes ranked by NormFinder.

Rank	Cold	Drought	Heat	Salt	High pH	Wounding	ABA	JA	SA	Total
**1**	*SKIP5*	*EF1beta*	*ACT*	*UBC15*	*UBC15*	*UBC2*	*CUL4*	*UBC17*	*HERC2*	*UBC15*
**Stability value**	0.076	0.009	0.097	0.033	0.066	0.036	0.056	0.075	0.032	0.154
**2**	*HERC2*	*UBC15*	*SKIP1*	*EF1beta*	*EF1beta*	*UBC15*	*UBC15*	*EF1beta*	*UBC17*	*HERC2*
**Stability value**	0.111	0.112	0.118	0.037	0.158	0.036	0.094	0.098	0.051	0.172
**3**	*UBC17*	*GAPDH*	*GAPDH*	*HERC2*	*SKIP5*	*UBC17*	*UBC17*	*UBC15*	*EF1beta*	*EF1beta*
**Stability value**	0.117	0.128	0.133	0.125	0.234	0.040	0.107	0.126	0.052	0.187
**4**	*CUL4*	*ACT*	*HERC2*	*UCH*	*HERC2*	*EF1beta*	*ACT*	*CUL4*	*UBC2*	*ACT*
**Stability value**	0.137	0.130	0.145	0.178	0.249	0.078	0.122	0.138	0.118	0.196
**5**	*UBC15*	*HERC2*	*UCH*	*SKIP1*	*UCH*	*CUL4*	*SNF2*	*UBC2*	*SKIP1*	*SKIP1*
**Stability value**	0.140	0.131	0.174	0.182	0.254	0.079	0.123	0.148	0.136	0.237
**6**	*UCH*	*SamDC*	*CUL4*	*CAP*	*UBC2*	*UCH*	*HERC2*	*SNF2*	*CUL4*	*CAP*
**Stability value**	0.173	0.144	0.185	0.211	0.263	0.097	0.124	0.149	0.150	0.240
**7**	*ACT*	*CAP*	*SNF2*	*GAPDH*	*CUL4*	*SKIP1*	*EF1beta*	*ACT*	*UBC15*	*UBC2*
**Stability value**	0.188	0.154	0.246	0.325	0.263	0.107	0.130	0.170	0.157	0.261
**8**	*GAPDH*	*UBC2*	*CAP*	*SNF2*	*ACT*	*ACT*	*UCH*	*SKIP1*	*UCH*	*GAPDH*
**Stability value**	0.189	0.159	0.259	0.336	0.263	0.110	0.138	0.186	0.169	0.268
**9**	*SKIP11*	*UBC17*	*UBC15*	*ACT*	*UBC17*	*HERC2*	*UBC2*	*UCH*	*SNF2*	*UCH*
**Stability value**	0.194	0.173	0.264	0.348	0.298	0.136	0.198	0.194	0.190	0.289
**10**	*SamDC*	*SKIP1*	*EF1beta*	*SKIP5*	*CAP*	*SNF2*	*CAP*	*HERC2*	*ACT*	*UBC17*
**Stability value**	0.212	0.234	0.308	0.387	0.325	0.139	0.200	0.203	0.237	0.296
**11**	*SNF2*	*UCH*	*UBC2*	*UBC2*	*GAPDH*	*CAP*	*SKIP1*	*GAPDH*	*SamDC*	*SNF2*
**Stability value**	0.220	0.270	0.317	0.445	0.349	0.153	0.242	0.224	0.254	0.299
**12**	*EF1beta*	*CUL4*	*UBC17*	*CUL4*	*SKIP1*	*SamDC*	*GAPDH*	*SKIP5*	*CAP*	*CUL4*
**Stability value**	0.233	0.299	0.333	0.499	0.350	0.266	0.257	0.259	0.260	0.309
**13**	*CAP*	*SKIP5*	*SamDC*	*UBC17*	*SNF2*	*SKIP5*	*SKIP5*	*SamDC*	*SKIP5*	*SamDC*
**Stability value**	0.235	0.328	0.346	0.530	0.397	0.334	0.291	0.346	0.284	0.354
**14**	*UBC2*	*SNF2*	*SKIP11*	*SamDC*	*SamDC*	*GAPDH*	*SamDC*	*CAP*	*GAPDH*	*SKIP5*
**Stability value**	0.256	0.381	0.556	0.542	0.471	0.351	0.330	0.351	0.301	0.371
**15**	*SKIP1*	*SKIP11*	*SKIP5*	*SKIP11*	*SKIP11*	*SKIP11*	*SKIP11*	*SKIP11*	*SKIP11*	*SKIP11*
**Stability value **	0.311	0.761	0.728	0.882	0.807	0.850	0.367	0.411	0.567	0.681

### Bestkeeper analysis of reference gene expression stability

As shown in Table 3, Bestkeeper ranked *UBC17* as the most stably expressed gene following cold, wounding, and ABA treatments, while *UCH* had the lowest SD value under drought and salt stresses. *GAPDH*, *SamDC*, *UBC2*, and *SKIP1* were ranked as the most stably expressed genes under heat, high pH, JA, and SA treatments, respectively. When all the samples were analyzed together, *UCH* was rated as the most stable gene with the lowest SD value.

**Table 3:** Stabilities of candidate reference genes ranked by Bestkeeper.

Rank	Cold	Drought	Heat	Salt	High pH	Wounding	ABA	JA	SA	total
**1**	*UBC17*	*UCH*	*GAPDH*	*UCH*	*SamDC*	*UBC17*	*UBC17*	*UBC2*	*SKIP1*	*UCH*
**SD**	0.11	0.32	0.46	0.35	0.65	0.16	0.08	0.19	0.11	0.41
**2**	*SKIP5*	*GAPDH*	*EF 1beta*	*HERC2*	*UCH*	*CUL4*	*CUL4*	*EF 1beta*	*UBC17*	*SamDC*
**SD**	0.13	0.47	0.48	0.37	0.76	0.22	0.11	0.21	0.12	0.52
**3**	*SamDC*	*SamDC*	*UBC17*	*UBC15*	*UBC15*	*ACT*	*HERC2*	*GAPDH*	*HERC2*	*SNF2*
**SD**	0.14	0.50	0.52	0.38	0.97	0.24	0.13	0.22	0.14	0.54
**4**	*ACT*	*UBC2*	*UCH*	*EF 1beta*	*HERC2*	*UBC2*	*UBC15*	*ACT*	*EF 1beta*	*ACT*
**SD**	0.16	0.55	0.54	0.41	1.00	0.25	0.13	0.23	0.15	0.54
**5**	*UBC15*	*SKIP5*	*ACT*	*SamDC*	*SKIP5*	*UBC15*	*ACT*	*UCH*	*UBC15*	*EF 1beta*
**SD**	0.18	0.55	0.57	0.42	1.01	0.25	0.14	0.24	0.15	0.55
**6**	*HERC2*	*ACT*	*HERC2*	*SKIP5*	*UBC2*	*EF 1beta*	*SNF2*	*UBC15*	*UBC2*	*SKIP5*
**SD**	0.20	0.56	0.57	0.43	1.01	0.28	0.16	0.24	0.19	0.59
**7**	*GAPDH*	*CAP*	*UBC2*	*CAP*	*GAPDH*	*SKIP1*	*CAP*	*UBC17*	*UCH*	*GAPDH*
**SD**	0.22	0.59	0.58	0.48	1.02	0.29	0.16	0.25	0.20	0.60
**8**	*SKIP11*	*EF 1beta*	*CUL4*	*SKIP1*	*SKIP1*	*SKIP5*	*UCH*	*CUL4*	*GAPDH*	*HERC2*
**SD**	0.24	0.61	0.58	0.48	1.02	0.29	0.16	0.29	0.22	0.61
**9**	*CUL4*	*SNF2*	*SKIP11*	*ACT*	*CUL4*	*UCH*	*EF 1beta*	*SKIP11*	*ACT*	*SKIP1*
**SD**	0.25	0.63	0.59	0.52	1.04	0.30	0.20	0.33	0.24	0.67
**10**	*UCH*	*SKIP1*	*UBC15*	*GAPDH*	*SNF2*	*SNF2*	*UBC2*	*SNF2*	*SNF2*	*UBC15*
**SD**	0.26	0.65	0.60	0.53	1.05	0.33	0.22	0.34	0.25	0.67
**11**	*SKIP1*	*UBC15*	*SNF2*	*UBC2*	*EF 1beta*	*HERC2*	*SKIP1*	*SKIP5*	*SamDC*	*UBC2*
**SD**	0.29	0.69	0.61	0.56	1.07	0.34	0.25	0.35	0.30	0.67
**12**	*SNF2*	*HERC2*	*SKIP1*	*SNF2*	*UBC17*	*CAP*	*GAPDH*	*SKIP1*	*CUL4*	*CAP*
**SD**	0.35	0.72	0.61	0.62	1.08	0.39	0.29	0.36	0.31	0.72
**13**	*UBC2*	*UBC17*	*CAP*	*UBC17*	*CAP*	*GAPDH*	*SKIP5*	*HERC2*	*SKIP5*	*UBC17*
**SD**	0.36	0.74	0.65	0.66	1.08	0.41	0.32	0.41	0.32	0.73
**14**	*CAP*	*CUL4*	*SamDC*	*CUL4*	*ACT*	*SamDC*	*SKIP11*	*SamDC*	*CAP*	*CUL4*
**SD**	0.37	0.89	0.72	0.72	1.09	0.41	0.34	0.42	0.36	0.75
**15**	*EF 1beta*	*SKIP11*	*SKIP5*	*SKIP11*	*SKIP11*	*SKIP11*	*SamDC*	*CAP*	*SKIP11*	*SKIP11*
**SD**	0.40	1.02	0.91	0.80	1.19	0.94	0.36	0.56	0.63	1.04

SD represents standard deviation

### RefFinder ranking of the most stable genes

RefFinder, which combines the geNorm, NormFinder, and BestKeeper outputs, was applied to generate a comprehensive final ranking of the candidate references. As shown in Table 4, *EF1beta* (drought and JA), *ACT* (heat), *SKIP5* (cold), *UBC15* (salt, high pH, and ABA), *UBC2* (wounding), and *HERC2* (SA) were ranked as the top stable genes under the different treatments. Across all samples, *EF1beta*, *HERC2*, and *UCH* were suggested as the three best reference genes for normalizing the expression of target genes.

**Table 4:** Stabilities of candidate reference genes ranked by RefFinder.

	Drought	Heat	Cold	Salt	High pH	Wounding	JA	ABA	SA	Total
**Best**	*EF1beta*	*ACT*	*SKIP5*	*UBC15*	*UBC15*	*UBC2*	*EF1beta*	*UBC15*	*HERC2*	*EF1beta*
	*UBC15*	*GAPDH*	*UBC17*	*EF1beta*	*HERC2*	*UBC15*	*UBC17*	*CUL4*	*UBC17*	*HERC2*
	*GAPDH*	*HERC2*	*HERC2*	*HERC2*	*EF1beta*	*UBC17*	*UBC15*	*UBC17*	*EF1beta*	*UCH*
	*HERC2*	*SKIP1*	*UBC15*	*UCH*	*CUL4*	*UCH*	*UBC2*	*EF1beta*	*SKIP1*	*ACT*
	*SamDC*	*UCH*	*ACT*	*SKIP1*	*UBC17*	*CUL4*	*CUL4*	*SNF2*	*UBC2*	*UBC15*
**Worst**	*SKIP11*	*SKIP5*	*SKIP1*	*SKIP11*	*SKIP11*	*SKIP11*	*CAP*	*SKIP11*	*SKIP11*	*SKIP11*

### Validation of candidate reference genes

**Fig 4:**
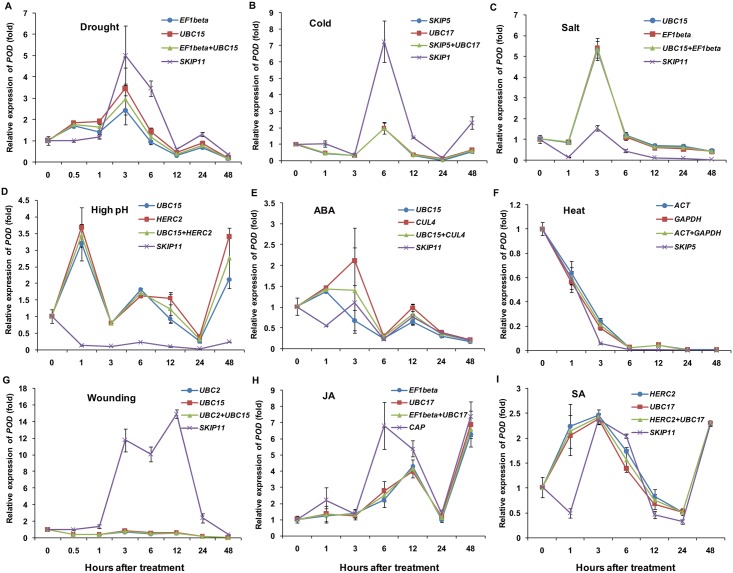
Validation of the most and least stable reference genes for the normalization of *SgPOD* expression. The most and least stable reference genes were selected for normalization following different stress treatments for 0, 1, 3, 6, 12, 24, and 48 h, with an additional 0.5-h time point for the drought and wounding treatments. (A) Drought. (B) Cold. (C) Salt. (D) High pH. (E) ABA. (F) Heat. (G) Wounding. (H) JA. (I) SA. The error bars represent the mean of three technical replicates ± SD.

**Fig 5:**
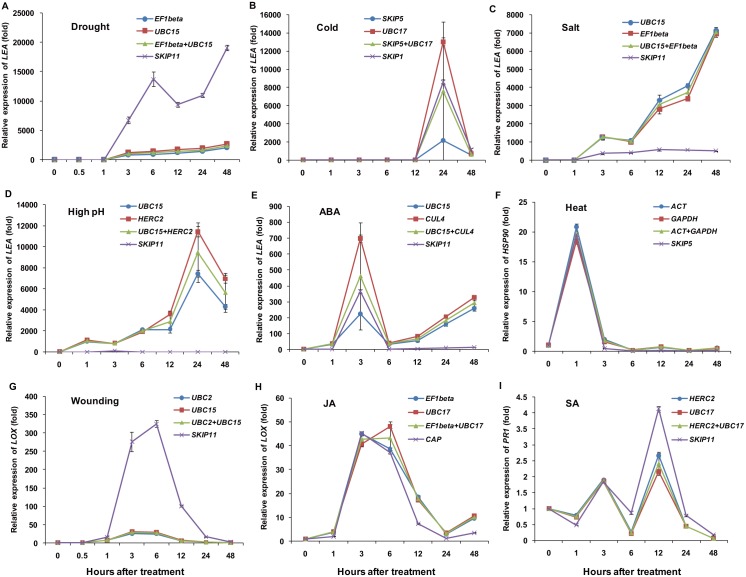
Validation of the most and least stable reference genes for the normalization of *SgLEA*, *SgLOX*, *SgHSP90*, and *SgPR1* expression. The most and least stable reference genes were selected for normalization following different stress treatments for 0, 1, 3, 6, 12, 24, and 48 h, with an additional 0.5-h time point for the drought and wounding treatments. (A–E) *SgLEA* expression under drought, cold, salt, high pH, and ABA treatments. (F) *SgHSP90* expression under heat treatment. (G and H) *SgLOX* expression under wounding and JA treatments. (I) *SgPR1* expression under SA treatment. The error bars represent the mean of three technical replicates ± SD.

To validate the utility of the candidate reference genes selected using RefFinder, the expression patterns of six target genes identified from RNA-Seq data, including *SgPOD*, *SgPAL*, *SgLEA*, *SgLOX*, *SgHSP90*, and *SgPR1*, in response to different treatments were examined. When normalization was performed using the two most stable genes under each treatment, *EF1beta* and *UBC15* (drought), *SKIP5* and *UBC17* (cold), *UBC15* and *EF1beta* (salt), *ACT* and *GAPDH* (heat), *UBC15* and *HERC2* (high pH), *UBC2* and *UBC15* (wounding), *EF1beta* and *UBC17* (JA), *UBC15* and *CUL4* (ABA), and *HERC2* and *UBC17* (SA), the expression of *SgPOD* was affected by different treatments (Fig 4), and peak points were observed at 3 h under drought, at 6 h under cold, at 3 h under salt, at 1 h under high pH, at 48 h under JA, and at 3 h under SA, whereas minimums were found at 24 h under heat and at 48 h under ABA and wounding treatments. It is worth noting that the transcripts of *SgLEA* were intensively induced by drought, cold, salt, high pH, and ABA treatments (Fig 5A–5E). Wounding and JA treatments caused transcript accumulation of *SgLOX* (Fig 5G and 5H), and heat stress increased the expression level of *SgHSP90* (Fig 5F). An increased transcript level of *SgPR1* was observed at 3 h after SA treatment compared with the control, and a peak point was observed at 12 h following a decrease in expression at 6 h (Fig 5I). Additionally, upregulated expression of *SgPAL* was observed after JA, high pH, and SA treatments, whereas *SgPAL* expression was downregulated by the other treatments (S2 Fig). Similar expression patterns were obtained using the most stable single genes and combinations of two genes for normalization under the above treatments, while different expression patterns were observed when using the least stable reference genes compared with the most stable genes for target gene normalization.

## Discussion

qRT-PCR combines reverse transcription (RT) with the quantitative real-time polymerase chain reaction (qPCR) [35]. The accuracy of qRT-PCR is influenced strongly by the stability of the reference gene chosen for normalization of gene expression data [22]; therefore, it is crucial to select stably expressed genes as internal references [13].

Base on previous transcriptome data [8], we selected 15 genes that exhibited relatively low levels of transcript variation after grazing stress and evaluated their expression stability under different stresses in *S*. *grandis*. Four statistical algorithms, geNorm, NormFinder, BestKeeper, and RefFinder, were used to evaluate the stability of these genes. All the genes displayed different expression patterns following the various treatments. Moreover, high stability levels were observed using geNorm analysis; all the M values were below the accepted threshold of 1.5 (Fig 2), which indicated that all the genes could be used as internal control genes for normalization. However, according to different algorithms and analytical procedures, several differences were observed in the ranking of the most stable genes, which was similar to the results for other species [19–21]. For wounding treatment in *S*. *grandis*, *UBC2* and *UBC15* were selected by both geNorm and NormFinder as the two most stable reference genes (Fig 2 and Table 2), but *UBC17* and *CUL4* were ranked top by Bestkeeper (Table 3); *UBC2*, *UBC15*, and *UBC17* were determined as the most stable by RefFinder’s comprehensive analysis (Table 4). Across all treatments under study, *UBC17*, *CUL4*, and *HERC2* for geNorm (Fig 2), *UBC15*, *HERC2*, and *EF1beta* for NormFinder (Table 2), and *UCH*, *SamDC*, and *SNF2* for Bestkeeper were considered the most suitable reference genes (Table 3). Overall, RefFinder recommended using *EF1beta*, *HERC2*, and *UCH* (Table 4).

Traditional reference genes, such as *ACT*, *GAPDH*, and *EF1α*, which are involved in primary metabolism or other cellular processes [19], as well as new candidate reference genes derived from public databases [12, 17, 22, 25, 26], have been validated to allow precise interpretation of the qRT-PCR results for specific experimental conditions. However, fluctuating expression of reference genes has been observed under certain experimental conditions. For example, *EF1α* was identified as the best reference gene under salinity and drought stresses in *Glycine max* [36]. Nevertheless, *EF1α* exhibited poor performance in leaves and roots during abiotic stress treatments [15]. In the present study, we also found that the most suitable reference genes varied under different environmental stimuli. The comprehensive combined analysis by RefFinder showed that the top three ranked genes included the traditional genes *EF1beta*, *ACT*, *UBC15*, *UBC2*, *GAPDH*, and *UBC17*, as well as the novel genes *UCH*, *SKIP5*, *HERC2*, and *CUL4* (Table 4).

Previously, *EF1beta* was identified as a stable reference gene in the developing seeds of the Tung Tree [37]. *EF1beta* has also been determined to be a good reference in soybean under various stresses [36]. Similarly, we found that *EF1beta* was in the three top ranked reference genes for drought, salt, high pH, JA, and SA treatments, as well as across all samples (Table 4). *ACT*, a traditional reference gene, is used commonly as an internal control to normalize target gene expression results from qRT-PCR in various species under different conditions. For instance, *ACT* was determined to be stably expressed during different floral developmental stages and different temperature treatments in Sweet Osmanthus (*Osmanthus fragrans Lour*.) [14]. In our study, *ACT* was identified as the best reference gene by the geNorm and RefFinder programs under heat stress (Fig 2 and Table 4).

Ubiquitination is related to post-translational modification of proteins, and three enzymes are involved in this cellular process: E1 (ubiquitin-activating enzymes), E2 (ubiquitin-conjugating enzymes), and E3 (ubiquitin ligases) [38]. Accumulated data indicate that ubiquitin-conjugating enzymes (UBC) exhibit stable expression in many experimental conditions; thus, they have been used widely as reference genes in different species [12, 14, 39]. Three *UBC* genes (*UBC15*, *UBC2*, and *UBC17*) were analyzed in this study. Notably, these *UBC* genes performed well under different treatments (excluding heat stress), especially when analyzed by geNorm and RefFinder (Fig 2 and Table 4). *UBC15* and *UBC17* exhibited better expression stability than *UBC2*. *HERC2*, a HECT domain E3 (ubiquitin ligases) encoding gene [40], also showed stable transcript levels under most experimental conditions. Additionally, the de-ubiquitinating protease UCH (ubiquitin carboxyl-terminal hydrolase) was the top ranked gene using Bestkeeper analysis under drought and salt treatments, as well as in the overall assessment (Table 3). Taken together, at least one of the ubiquitin-related genes was ranked in the top three stable genes (Fig 2, Tables 3 and 4). These results indicated the *UBC* genes could be used more often as an internal control for normalizing gene expression under various stress treatments. SKIP, a spliceosome component and transcriptional activator, is conserved in eukaryotes and has diverse functions in plant development associated with responses to environmental changes [41]. Among the three SKIP encoding genes examined in this study, *SKIP5* was ranked as the most stable gene under cold stress using geNorm (Fig 2), NormFinder (Table 2), and RefFinder (Table 4), and *SKIP1* was recommended as the best gene under JA treatment by Bestkeeper analysis (Table 3). Conversely, *SKIP11* was ranked the least stable reference gene by the different programs (Fig 2 and Tables 2–4), except for heat and cold stresses by geNorm and NormFinder, heat, cold, ABA, and JA treatments by Bestkeeper, and heat, cold, and JA treatments by RefFinder. These results indicate that *SKIP11* is the least suitable gene for use as an internal control in *S*. *grandis* under various environmental stimuli.

The effectiveness of the most stable reference genes selected using RefFinder was evaluated by normalization of six target genes in samples under different stress conditions. Peroxidases [POD/POX, EC:1.11.1.7] are heme-containing glycoproteins that oxidize a wide range of hydrogen donors, accompanied by the accumulation of H_2_O_2_ [42]. Many peroxidase isoenzymes are found in higher plants and they play roles in plant defense, growth, and development [42]. Guaiacol peroxidase (GPX, EC 1.11.1.7) [43], which is generally considered a stress-related enzyme, is involved in plant defense against various stresses, and the activity of GPX is thought to be induced by diverse environmental stimuli [44], such as drought, salinity, and chilling [43, 45, 46]. In the present work, the expression of a peroxidase encoding gene was detected under the different treatments (Fig 4), and the changes in its transcript levels suggested that peroxidase plays an important role in *S*. *grandis* defense against stressful conditions. The late embryogenesis abundant protein encoding gene *LEA* has been widely reported to participate in responses to drought, cold, and salt stresses, and ABA treatment, and plays an important role in plant tolerance to abiotic stress [47–51]. The significantly increased expression of *SgLEA* under drought, cold, salt, high pH and ABA treatments (Fig 5A–5E) implies that *SgLEA* participates in *S*. *grandis* resistance responses against stresses. The expression level of *SgLOX* was elevated by wounding and JA treatments (Fig 5G and 5H), and *SgHSP90* expression was enhanced by heat treatment (Fig 5F); the induction of these genes was in accordance with previously reported expression patterns of *LOX2* [52–54] and *HSP90* [55–57]. Additionally, a pathogenesis-related protein 1 encoding gene, *SgPR1*, which is widely accepted as an SA marker gene [58] and is induced by SA accumulation [59], was selected to assess the utility of the identified reference genes under SA treatment. As a critical defense signaling molecule, SA accumulation is associated with enhanced resistance to pathogen infection [60]. The changes in the transcript level of *SgPR1* after SA treatment indicated that *SgPR1* could be used as a molecular marker for disease resistance responses in *S*. *grandis* (Fig 5I). However, when the target gene normalization was performed with the least stable genes, the expression patterns varied in comparison with most stable reference genes. Taken together, these results suggest that appropriate selection of reference genes is necessary for the normalization of target gene expression to ensure the accuracy of qRT-PCR data.

## Conclusions

This study comprehensively evaluated the utility of 15 potential reference genes for quantifying transcript levels in *S*. *grandis* exposed to diverse experimental conditions. The stable reference genes identified in this work will help to improve the accuracy of gene expression analysis and will aid the investigation of stress-response genes and the molecular mechanism of stress tolerance in this species.

## Supporting Information

S1 Fig Primer specificity and amplicon size for the candidate reference genes. (TIF)

S2 Fig Validation of the most and least stable reference genes for the normalization of *SgPAL* expression under different treatments. (TIF)

S1 Text Sequences of candidate reference genes. (TXT)
