# Supplementary figures and images for: Selection of Reference Genes for qRT-PCR Analysis of Gene Expression in Stipa grandis during Environmental Stresses
Source: PLoS One. 2017 Jan 5;12(1):e0169465. doi: 10.1371/journal.pone.0169465 (PMC5215803; doi:10.1371/journal.pone.0169465)

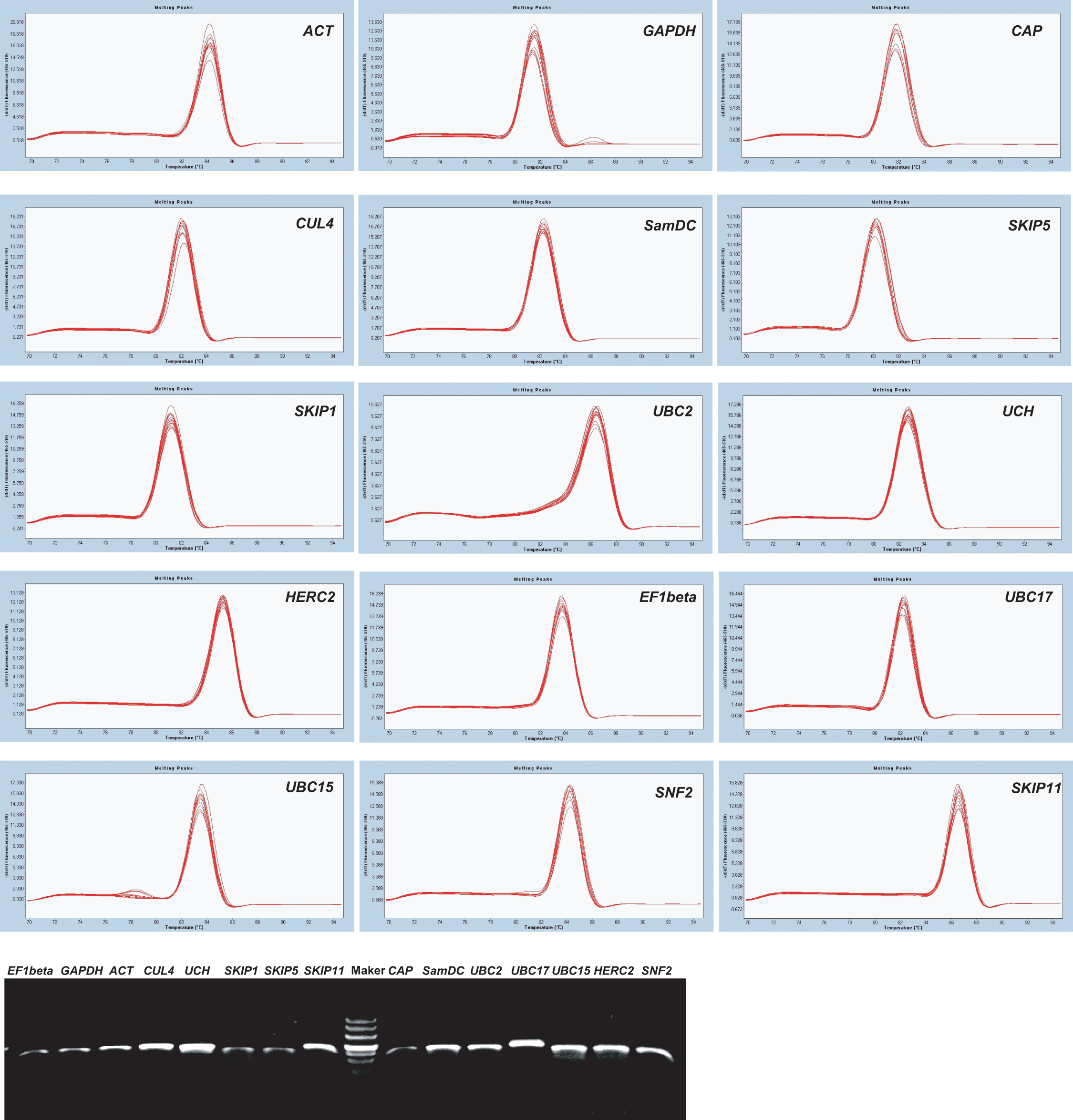

Supplement: S1 Fig — (TIF) [file pone.0169465.s001.tif]

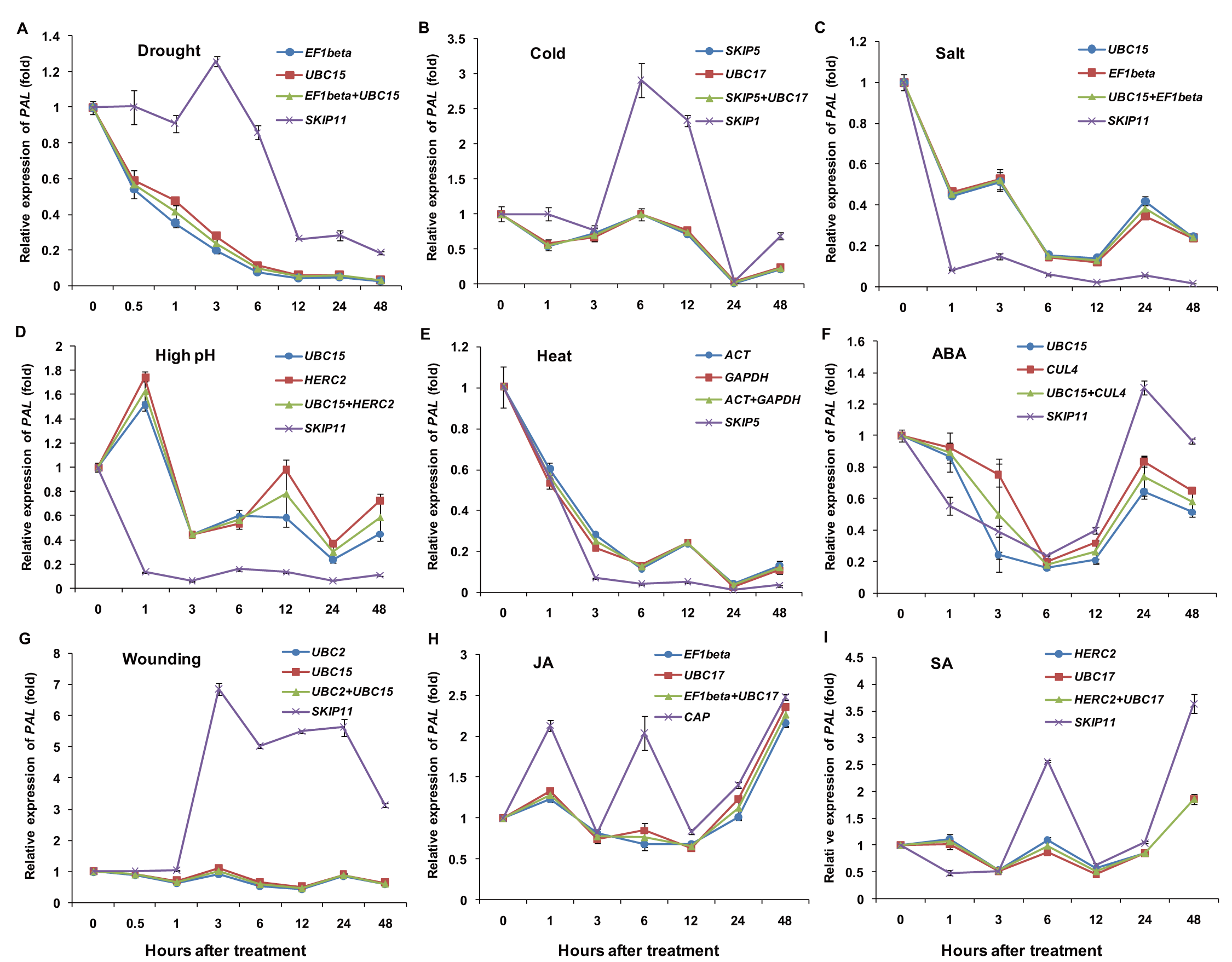

Supplement: S2 Fig — (TIF) [file pone.0169465.s002.tif]
